# Exploring the Microbial Landscape of Neonatal Skin Flora: A Comprehensive Review

**DOI:** 10.7759/cureus.52972

**Published:** 2024-01-26

**Authors:** Aditya Jain, Revat J Meshram, Sham Lohiya, Ankita Patel, Divyanshi Kaplish

**Affiliations:** 1 Pediatrics, Jawaharlal Nehru Medical College, Datta Meghe Institute of Higher Education and Research, Wardha, IND

**Keywords:** public health implications, gestational age, mode of delivery, immune development, microbial colonization, neonatal skin microbiome

## Abstract

This comprehensive review explores the intricate landscape of the neonatal skin microbiome, shedding light on its dynamic composition, developmental nuances, and influential factors. The neonatal period represents a critical window during which microbial colonization significantly impacts local skin health and the foundational development of the immune system. Factors such as mode of delivery and gestational age underscore the vulnerability of neonates to disruptions in microbial establishment. Key findings emphasize the broader systemic implications of the neonatal skin microbiome, extending beyond immediate health outcomes to influence susceptibility to infections, allergies, and immune-related disorders. This review advocates for a paradigm shift in neonatal care, proposing strategies to preserve and promote a healthy skin microbiome for long-term health benefits. The implications of this research extend to public health, where interventions targeting the neonatal skin microbiome could potentially mitigate diseases originating in early life. As we navigate the intersection of research and practical applications, bridging the gap between knowledge and implementation becomes imperative for translating these findings into evidence-based practices and improving neonatal well-being on a broader scale.

## Introduction and background

Neonates, particularly in the early stages of life, embark on a journey of microbial colonization that profoundly shapes their developing physiology. The human microbiome, comprising trillions of microorganisms residing on and within the body, plays a pivotal role in various aspects of health. Among these microbial ecosystems, the neonatal skin microbiome emerges as a critical interface between the external environment and the newborn's rapidly evolving physiology [1].

Establishing a diverse and balanced microbial community in neonates is a crucial event with far-reaching implications for both short-term well-being and long-term health outcomes. Early microbial exposure is known to influence the development and maturation of the immune system, impacting susceptibility to infections, allergies, and other immune-related disorders. Understanding the intricacies of microbial colonization in neonates becomes paramount in unraveling the complex interplay between the developing host and its microbial inhabitants [2].

While various body sites host unique microbial communities, the skin, the body's largest organ, serves as an intricate ecosystem harboring diverse microorganisms. Neonatal skin, in particular, undergoes a dynamic colonization process, establishing the foundation for the skin microbiome that will persist throughout an individual's life. The significance of this early colonization extends beyond mere symbiosis, influencing skin health, barrier function, and even systemic immune responses. As such, investigating the neonatal skin flora becomes imperative in comprehending the broader implications for individual health trajectories [3].

This review aims to comprehensively explore the neonatal skin microbiome, delving into its composition, developmental nuances, and the many factors shaping its landscape. By synthesizing current knowledge, this review sheds light on the intricate relationships between neonates and their skin flora, emphasizing the pivotal role that microbial communities play in the early stages of human development. Through a critical analysis of existing literature, we aim to not only elucidate the current understanding but also identify gaps in knowledge and suggest future research directions. Ultimately, this review aspires to contribute to the growing knowledge surrounding neonatal health, with implications for clinical practice, interventions, and public health strategies.

## Review

Neonatal skin microbiome

Composition

Overview of the predominant microbial species: The predominant microbial species present on neonatal skin exhibit variability influenced by age, mode of delivery, environmental exposures, and gestational age. Scientific investigations reveal that neonatal skin initially harbors bacteria from the mother's vaginal or skin microbiota, with the composition of the skin microbiome changing over time [4,5]. Major bacterial species on neonatal skin include *Staphylococcus aureus*, *Streptococcus pyogenes*, *Pseudomonas*, *Acinetobacter*, *Aeribacillus*, *Phylobacterium*, and *Serratia* [6]. The distribution of these species varies across different body sites, with some being more prevalent at the floor level of the neonatal environment and others exhibiting a higher abundance with increased wall height [4]. Environmental factors, such as antibiotic use and hygiene practices, further influence the neonatal skin microbiome's composition, potentially impacting health outcomes [7]. The neonatal skin microbiome is a sophisticated and dynamic system shaped by diverse factors, necessitating additional research to comprehensively understand its implications for infant health and immune development [5].

Factors influencing microbial diversity: Microbial diversity is intricately shaped by a multitude of factors, encompassing spatial and resource considerations, nutrient availability, distances to potential source pools, environmental heterogeneity, pH levels, climate, soil type, vegetation type, land-use patterns, and altitude [8,9]. Assessing the diversity of soil microbial communities involves characterizing their community-level physiological profiles (CLPPs), offering a swift method to gauge functional diversity [10]. Moreover, in neonates, microbial diversity is subject to influences such as delivery mode, gestational age, postnatal age, and environmental exposures [11]. The order and timing of colonization events are pivotal in determining how microbial strains interact, potentially yielding long-term consequences for microbiome composition [8]. In essence, microbial diversity emerges as a complex and dynamic system influenced by various factors, necessitating further research to comprehensively grasp its implications for health and environmental outcomes.

Developmental Changes

Evolution of skin microbiota during the neonatal period: The neonatal period represents a dynamic phase characterized by significant microbial colonization and immune development, particularly within the evolving landscape of the skin. During infancy, the skin undergoes pronounced structural and functional changes, influencing the development of the skin microbiome. These alterations encompass shifts in pH, water content, transepidermal water loss, and sebum production. The initial colonization of the skin microbiota occurs shortly after birth, demonstrating a preference for maternal strains, and undergoes frequent strain replacements over time. Notably, the skin microbiota composition in human infants and children differs distinctly from that of adults, with birth mode influencing the bacterial genera present on the skin during the first hours to days of life. Further restructuring of the relative abundance of skin microbial species occurs during puberty, marked by increased hormone levels stimulating additional sebum production from sebaceous glands. Exploring the factors that mold and sustain the skin microbiota during the neonatal period holds the potential to unveil strategies for therapeutic manipulation of the microbiota, thereby enhancing neonatal health outcomes [5,12-15].

Dynamic interactions between host and microbes: The dynamic interactions between hosts and microbes are intricate and multifaceted, playing a pivotal role in shaping aging dynamics and fostering mutualistic relationships through the coevolution of microbes and multicellular hosts [16]. When studying bacterial communities, comprehending these interactions requires focusing on metabolic modeling, considering the constant processes of cell division and death [17]. In conditions like cystic fibrosis, the interplay between host-microbe and microbe-microbe interactions becomes crucial, influencing changes in the respiratory microbiota through microbial interactions with the host nutritional environment, immune response, and other microbes [18]. In insects, a profound symbiotic association with their gut microflora has evolved into an essential and intricate evolutionary relationship [19]. Furthermore, dynamic interactions within host-associated microbiota have been found to contribute to tumor formation in basal metazoans like *Hydra*, underscoring the significance of understanding these complex relationships across various contexts [20]. These examples underscore the importance of delving into the complex and dynamic interactions between hosts and microbes to unravel their profound implications in diverse biological scenarios.

Factors influencing the neonatal skin microbiome

Mode of Delivery

Differences in skin flora between vaginally and cesarean-delivered infants: Research findings indicate notable differences in the skin flora of neonates depending on the mode of delivery, with distinctions between those born vaginally and via cesarean section. Neonates born vaginally tend to be predominantly colonized by *Lactobacillus*, while infants delivered through cesarean sections exhibit a mixture of potentially pathogenic bacteria such as* Staphylococcus* and *Acinetobacter*. This suggests that cesarean-delivered infants are colonized with skin flora rather than the typical vaginal bacteria associated with traditional deliveries [21,22]. The impact of the mode of delivery extends beyond the skin, affecting the bacterial community in both the newborn gut and skin, resulting in variations in the types of microorganisms acquired based on the mode of birth [21,22]. Despite these observed differences, the long-term consequences of the initial skin colonization modes in neonates remain unknown, emphasizing the need for further research to comprehensively understand the potential implications of these early microbial acquisitions on long-term health outcomes [21,22].

Impact on long-term health outcomes: The impact of the neonatal skin microbiome on long-term health outcomes has garnered increasing attention, with research highlighting its pivotal role in skin health, immune regulation, and defense against pathogenic infections. Dysbiosis, characterized by an imbalance in the skin microbiome, has been linked to various skin diseases and conditions, underscoring the importance of maintaining a healthy skin microbiota [5,23,24]. The skin microbiome actively develops the innate immune system, preserving skin homeostasis and fostering colonization resistance against pathogenic infections in healthy skin with a balanced microbiota [25]. Additionally, interactions between the skin microbiota and the host's immune system are crucial, as dysbiosis can lead to the dysregulation of bodily functions and contribute to the onset of various diseases [25]. While specific long-term health outcomes associated with the neonatal skin microbiome are still being unveiled, it is evident that sustaining a healthy skin microbiota is essential for overall skin health and immune function. Ongoing research is imperative to fully comprehend the long-term implications of the neonatal skin microbiome on human health [26].

Gestational Age

Influence of preterm birth on skin microbiota: The gestational age of preterm infants significantly shapes the development of their skin microbiota. Numerous studies have underscored the correlation between gestational age and skin structure and function maturation, consequently influencing the cutaneous microbiome of preterm infants [12,27,13]. Notably, the skin microbiota in preterm infants exhibits considerable variation across individuals and by body site, reflecting disparities in environmental exposures and infant developmental stages [13]. Factors such as mode of delivery, environmental exposures, and maternal transmission further contribute to the diversity of the skin microbiota in preterm infants [28]. A comprehensive understanding of the dynamics of the skin microbiota in this vulnerable population is essential for identifying potential clinical implications and developing targeted therapeutic strategies to support the delicate skin microbiome of preterm infants [15,27].

Implications for immune system development: The development of the skin microbiota in preterm infants is intricately intertwined with the maturation of their immune system. This microbiota exhibits variability across individuals, body sites, and developmental stages, reflecting diverse environmental exposures and infant development. Notably, alterations to the microbiota during the neonatal immunity window in preterm infants may have consequential effects on immune system development, including innate immune tolerization and reprogramming. Research indicates distinct initial responses between preterm and full-term infant immune systems, converging after three months. The evolving neonatal skin microbiome appears to be influenced by factors such as neonatal skin characteristics, immune system development, and environmental variables like delivery mode, feeding method, and skin-to-skin contact. A profound understanding of the development of the skin microbiota in preterm infants is imperative for identifying potential clinical implications and formulating targeted therapeutic strategies to support the delicate skin microbiome of these vulnerable infants [12,13,15,28,29].

Role of neonatal skin microbiome in health and disease

Skin Barrier Function

Microbial contributions to skin integrity: The skin microbiome plays a pivotal role in upholding the integrity and barrier function of the skin, the body's largest organ and primary defense against environmental elements. Comprising millions of bacteria, fungi, and viruses, the skin microbiome significantly influences the skin barrier function [5,30]. The commensal microbiome is indispensable in maintaining the skin barrier through its involvement in physical, chemical, and immunological processes [30]. Moreover, the skin microbiota has been associated with critical aspects of barrier function, including the regulation of skin epidermal differentiation and the enhancement of wound healing [31]. Specific microbial populations, such as *Staphylococcus epidermidis*, contribute to skin barrier integrity by producing sphingomyelinase and aiding the host in ceramide production [32]. Thus, the skin microbiome is vital in safeguarding the integrity and functionality of the skin barrier, a prerequisite for shielding the body from pathogens and environmental stressors.

Protection against pathogens: The skin is a vital barrier, shielding the body from harmful microbes. Its structural composition and inherent antimicrobial properties not only provide a robust defense against external pathogens but also accommodate the presence of normal flora bacteria and fungi. The skin's innate immune system, complemented by specialized immune cells within the skin tissue, is pivotal in repelling microbial invaders and combating pathogens [33-35]. Furthermore, the skin microbiome, encompassing bacteria, fungi, and viruses, contributes to pathogen protection through the production of antimicrobial compounds and involvement in regulating the skin's epidermal differentiation and wound healing processes [31]. Thus, the skin and its associated microbiome preserve their defense mechanisms against harmful microbes.

Immunomodulation

Interaction between skin microbes and the immune system: The interplay between the skin microbiota and the immune system is critical to upholding skin function and regional homeostatic immunity. Engaging in intricate crosstalk with the host, the skin microbiota influences critical aspects such as establishing immunological tolerance, antimicrobial production, wound healing, barrier function, and the regulation of skin cell function. Nevertheless, pathogens and pathobionts within the skin microbiota can lead to diseases linked to various skin conditions. The composition of the skin microbiota is subject to the influence of diverse host factors and environmental elements, including age, sex, hormones, lifestyle, topical skin products, antibiotics, environment, hygiene, diet, and drugs [36]. Comprising a complex network of keratinocytes and immune cells, including skin-specific antigen-presenting cells, the skin immune system is the primary defense against infections. The delicate balance in the immune-microbial interaction is crucial, as even slight disruptions to this network can trigger inflammatory responses [37]. Research indicates that both the skin and intestine have evolved symbiotic relationships with commensal microbes, emphasizing the significance of their respective microbiota in maintaining epithelial homeostasis and overall health. Supporting a vast diversity and abundance of microbiota, the skin and intestine play pivotal roles in protective strategies, preventing pathogen entry, and facilitating tissue repair [38].

Implications for allergic and autoimmune diseases: Accumulating evidence underscores the pivotal role of the microbiota in the onset and progression of allergic and autoimmune diseases. Investigations reveal that skin and gut microbiota modifications can exert adverse effects on the immune system, leading to the development of autoimmune and allergic conditions, including atopic dermatitis, allergic rhinitis, asthma, and food allergies [39-41]. The hygiene hypothesis posits that changes in lifestyle, particularly in industrialized nations, resulting in a reduction in infectious exposure, may contribute to the escalating prevalence of autoimmune and allergic diseases [42]. Research further illuminates the role of the skin microbiota in the pathogenesis of autoimmune skin disorders through the influence of metabolites and recall immune responses [43]. Notably, bacterial extracts, such as OM-85, have demonstrated efficacy in various experimental models and clinical settings, presenting novel therapeutic perspectives for preventing autoimmune and allergic diseases [42]. These findings underscore the intricate relationship between the microbiota and immune dysregulation, offering promising avenues for therapeutic interventions in autoimmune and allergic diseases.

Methods of studying neonatal skin microbiota

Sampling Techniques

Swabbing, scraping, and other non-invasive methods: Various sampling methods are commonly employed to harvest the skin microbiota, with skin swabbing, tape-stripping, and punch biopsy being the primary techniques. Skin swabbing is one of the most frequently used methods because it is quick, simple, and non-invasive, making it well-suited for large-scale skin sampling. The process involves utilizing a sterile swab pre-moistened in a solution, typically 0.9% sodium chloride with 0.1% Tween. This method's preference arises from its non-invasiveness and capacity to sample extensive skin areas. Tape-stripping and punch biopsy, employed for skin microbiota sampling, are comparatively more invasive and less commonly utilized for routine sampling [44]. When studying neonatal skin microbiota, skin swab samples are gathered from infant skin sites, including the forehead, posterior auricular scalp, periumbilical region, inguinal folds, upper thighs, and oral cavity. These samples are then transported to the laboratory for further analysis, which may involve DNA extraction and sequencing to elucidate the neonatal skin microbiota's microbial diversity and composition [12,45]. Overall, non-invasive methods such as skin swabbing are favored for studying the neonatal skin microbiota due to their simplicity, non-invasiveness, and suitability for large-scale sampling. These techniques have significantly contributed to advancing our understanding of the neonatal skin microbiota and its potential implications for infant health.

Challenges and limitations: Exploring the human skin microbiome, including the neonatal skin microbiota, confronts several challenges and limitations. A primary obstacle lies in the underestimated diversity of the skin microbiota, primarily attributed to the biases introduced by conventional culture techniques. Addressing this challenge necessitates the adoption of novel sequencing technologies, although achieving standardized methodologies is imperative to draw robust conclusions regarding the most effective innovation processes [46]. Careful attention to each step of the research protocol is another critical challenge, encompassing considerations such as DNA extraction methods, library construction, sequencing procedures, and subsequent data analysis. This meticulous approach becomes particularly crucial when employing non-invasive sampling methods like skin swabbing, which may introduce contaminants, potentially affecting the accuracy of the results [46]. Moreover, the composition of the skin microbiota proves to be multifaceted, influenced by factors such as gestational age, postnatal age, body size, and environmental exposures, complicating the derivation of definitive conclusions about neonatal skin microbiota. The long-term consequences of the initial skin colonization modes in neonates remain unknown, emphasizing the necessity for further research to comprehend the enduring impact of early microbial acquisitions on health. In summary, the challenges and limitations of studying neonatal skin microbiota encompass its diverse composition, the meticulous attention required throughout the research process, and the intricate factors influencing its composition. Despite these challenges, ongoing advancements, including novel sequencing technologies and refined assessment tools such as 3D skin models, hold promise for enhancing our comprehension of the neonatal skin microbiota and its potential implications for infant health [46].

Next-Generation Sequencing

High-throughput techniques for microbial identification: Next-generation sequencing (NGS), or high-throughput sequencing (HTS), is a robust microbial identification tool. The NGS platforms excel at generating substantial data volumes in a single run, enabling unparalleled throughput and unbiased sequencing without prior knowledge of a sample's complete DNA. The NGS has revolutionized microbial studies widely employed for de novo genome sequencing, resequencing, targeted amplicon sequencing, genotyping, RNA-seq on low-complexity transcriptomes, and metagenomics [47,48]. The precision and speed of NGS have elevated it to a pivotal technology for clinical pathogen detection, facilitating swift, accurate, and specific identification of clinical specimens [49]. Additionally, NGS has proven valuable in studying fungal pathogens [49]. Integrating various NGS and third-generation sequencing (TGS) technologies into microbiome research has significantly contributed to the field, unraveling new insights [50]. Nevertheless, it's important to note that the initial capital investment and cost per experiment remain relatively high despite a substantial reduction in the price per information unit (nucleotide) compared to first-generation sequencing [47].

Advances in Understanding Microbial Communities

Machine learning and microbiome analysis: The prevailing microbiome analysis methods heavily rely on 'omics' approaches, particularly metagenomics involving 16S rRNA gene amplicon sequencing from community DNA samples [51]. Researchers are increasingly employing machine learning techniques to analyze the resulting data to discern patterns and relationships within microbial communities, enhancing our ability to decipher the complexities of microbiome dynamics.

Synthetic microbial communities: Ongoing research endeavors aim to elucidate the underlying mechanisms governing synthetic microbial communities' formation, maintenance, and evolution [52]. These artificially created communities offer a valuable tool for investigating fundamental biological questions spanning molecular biology, cellular/organismal biology, ecology, and evolution.

Human microbiome: The significant strides in genome sequencing have provided a deeper understanding of the composition and function of the human microbiome [53]. Given its dependence on structure and diversity, which vary extensively among individuals and are influenced by various factors, researchers are exploring the potential of personalized medicine grounded in an individual's unique microbiome.

Urinary tract infection: Advances in microbial community analysis within the human body have identified a resident microbial community in the urinary tract [53]. Ongoing investigations into the role of the urinary microbiome in urinary tract infection (UTI) and recurrent UTI (rUTI) aim to yield novel diagnostic and treatment strategies.

Environmental sampling: Technological advancements have empowered researchers to study microbial communities in diverse environments, including the walls of operating rooms (ORs) [4]. These studies unveil the bacterial composition and the impact of human activities on the microbiome, offering insights into the dynamics of microbial populations. Recent advances in understanding microbial communities have paved the way for exploring microbial populations' structure, function, and dynamics in various environments, ranging from the human body to natural ecosystems. These breakthroughs can potentially deepen our understanding of the role of microbes in health, disease, and ecosystem functioning [51-54].

Clinical applications and interventions

Probiotics and Prebiotics

Potential benefits for neonatal skin health: The neonatal skin microbiota plays a pivotal role in shaping the development of the infant's immune system and overall health. In the early stages, neonatal skin is initially colonized by microorganisms in the immediate environment, such as those in the NICU or hospital room, with a notable inclination towards maternal strains. The ongoing investigation focuses on identifying environmental reservoirs of bacteria, their impact on the skin microbiota, and the mechanisms facilitating transmission during early life. Gaining a comprehensive understanding of the dynamics of the skin microbiota during this critical period may unveil strategies to manipulate it for therapeutic purposes, potentially enhancing neonatal health outcomes [13,15]. In neonatal health, prebiotics and probiotics have garnered attention for their potential clinical applications in maintaining gut health and addressing specific health issues. Prebiotics, known for their ability to nourish the intestinal microbiota, produce short-chain fatty acids upon degradation, which are released into the bloodstream, influencing overall health. On the other hand, probiotics consist of live, nonpathogenic microorganisms and have been utilized in treating both gastrointestinal (GI) and non-GI medical conditions. While robust evidence supports the use of probiotics in treating acute diarrhea and pouchitis, the data regarding their efficacy for non-GI-associated illnesses often presents conflicting outcomes [7,55]. Despite the wealth of research on the benefits of prebiotics and probiotics for gut health, more attention should be given to their potential advantages for neonatal skin health. Nevertheless, existing studies indicate that probiotics can shield epidermal keratinocytes from Staphylococcus aureus-induced cell death, and prebiotics can modulate the skin microbiota by selectively stimulating the growth and activity of specific bacteria in the colon, thereby potentially enhancing overall host health [55]. Further research is imperative to fully grasp the potential benefits of prebiotics and probiotics for neonatal skin health.

Safety considerations: Ensuring the safety of probiotics, especially in vulnerable populations such as newborns and preterm infants, is a crucial aspect that demands careful consideration. While existing studies on infants and probiotics generally suggest their safety for healthy infants, a significant gap remains in the research on this subject. It is vital to recognize that not all probiotics are identical, and the FDA categorizes them as supplements. Consequently, there is no standardized protocol for administering probiotics, particularly to infants. Therefore, it is advisable to consult a pediatrician before introducing probiotics to infants, particularly those with weakened immune systems, underlying health issues, or those born prematurely, as adverse reactions, including infections, may occur [56]. Moreover, a 2023 perspective on probiotic safety underscores the necessity to establish standards for purity, potency, and the systematic tracking and reporting of adverse events related to probiotic products. Long-term studies specifically designed to demonstrate probiotic safety in at-risk populations, such as newborns, especially preterm infants, are notably limited. While the positive effects of probiotics in these groups are documented, individuals with compromised immune systems might be at an elevated risk for adverse events associated with probiotic use [57]. Hence, ensuring the safety of probiotics in vulnerable populations, including newborns and preterm infants, demands further research and meticulous consideration.

Personalized Medicine

Tailoring interventions based on individual skin microbiota: The skin microbiota exhibits considerable variability among individuals and is significantly influenced by extrinsic factors like cosmetics, environmental conditions, and antibacterial agents [58]. Extensive research demonstrates that the composition of the skin microbiome undergoes dynamic changes throughout an individual's lifetime, responding to significant life events and environmental influences [59]. The skin microbiota comprises microorganisms that exhibit variations based on the microenvironment dictated by anatomical location and surrounding factors [60]. While the microbial communities on the skin generally maintain stability over time, early-life microbial acquisitions may exert enduring effects on health outcomes [15]. Researchers are investigating bacteriotherapy to manipulate the skin microbiota by exploring novel therapeutic avenues. This innovative approach involves using specific bacterial strains to modulate the composition and function of the skin microbiota, aiming to enhance outcomes in inflammatory skin diseases [60]. Although navigating the field of bacteriotherapy for skin diseases poses challenges, it holds significant promise for developing personalized interventions tailored to an individual's unique skin microbiota. The skin microbiota, influenced by a myriad of extrinsic factors, undergoes dynamic changes over a person's lifespan. Bacteriotherapy emerges as a hopeful strategy, leveraging specific bacterial strains to target the skin microbiota for personalized interventions to preserve skin health and address inflammatory skin conditions.

Future Directions for Targeted Therapies

Tumor-agnostic approaches: The landscape of targeted oncology is evolving towards a tumor-agnostic paradigm, where treatment decisions hinge on specific genetic mutations rather than the tissue of origin. This approach exhibits promise for enhancing treatment outcomes across various cancer types, marking a significant advancement in precision medicine [61].

Molecular imaging: The exploration of molecular imaging in targeted therapies is gaining traction to optimize dosing schedules, determine therapeutic regimens, and monitor treatment responses. This approach can potentially facilitate timely adjustments in treatment protocols, particularly in scenarios involving therapy resistance [62].

Personalized medicine and genetic makeup: The trajectory of targeted therapies is intricately linked to personalized medicine, rooted in understanding each individual's genetic makeup. This revolutionary approach aims to tailor healthcare interventions based on the unique genetic characteristics of each person, promising improved disease prevention, precise diagnoses, and the delivery of safer, more effective treatments [63].

Expanding targeted therapy options: Current research and clinical trials are dedicated to identifying novel molecular targets and developing innovative targeted agents. These endeavors are focused on broadening the spectrum of cancers that can be effectively treated with targeted therapies, fostering optimism for enhanced patient outcomes, and alleviating the burden of these diseases [64].

Overcoming limitations and challenges: Despite significant progress, challenges persist in targeted therapies, including the limited eligibility of patients and the potential for resistance. Future directions involve addressing these challenges through ongoing research, the development of new therapeutic agents, and exploring combination therapies [65]. The future of targeted therapies, particularly in cancer treatment, is marked by a transition towards personalized and tumor-agnostic approaches, the integration of molecular imaging, continuous efforts to diversify targeted therapy options, and a commitment to overcoming existing limitations. These developments hold the potential to significantly enhance treatment outcomes and mitigate the impact of cancer and other diseases.

## Conclusions

Exploring the neonatal skin microbiome reveals pivotal insights into the complex interplay between neonates and their microbial communities. The dynamic composition and developmental changes of the skin microbiome underscore its significant influence on local skin health and the foundational development of the immune system. Factors such as mode of delivery and gestational age further highlight neonates' vulnerability to microbial colonization perturbations, emphasizing the critical nature of the early establishment of these microbial communities. Recognizing the far-reaching implications of microbial colonization for neonatal health, this review advocates for a paradigm shift in neonatal care. Strategies aimed at preserving and promoting a healthy skin microbiome hold the potential to prevent infections, allergies, and other immune-related disorders. Beyond individual health, the implications extend to public health, with interventions targeting the neonatal skin microbiome contributing to mitigating diseases in early life. As we move forward, translating these findings into evidence-based practices is essential for developing personalized healthcare approaches, ultimately improving the well-being of neonates and shaping healthier communities.
